# Microglia specific alternative splicing alterations in multiple sclerosis

**DOI:** 10.18632/aging.206045

**Published:** 2024-08-07

**Authors:** Caiyun Qi, Honglei Ren, Yong Fan

**Affiliations:** 1 Department of Obstetrics and Gynecology; Guangdong Provincial Key Laboratory of Major Obstetric Diseases; Guangdong Provincial Clinical Research Center for Obstetrics and Gynecology; Guangdong-Hong Kong-Macao Greater Bay Area Higher Education Joint Laboratory of Maternal-Fetal Medicine; The Third Affiliated Hospital, Guangzhou Medical University, Guangzhou, China; 2 Department of Neurology, Tianjin Neurological Institute, Tianjin Institute of Immunology, State Key Laboratory of Experimental Hematology, Haihe Laboratory of Cell Ecosystem, Tianjin Medical University General Hospital, Tianjin, China

**Keywords:** multiple sclerosis, alternative splicing, skipping exon, mutually exclusive exons, retained intron

## Abstract

Several aberrant alternative splicing (AS) events and their regulatory mechanisms are widely recognized in multiple sclerosis (MS). Yet the cell-type specific AS events have not been extensively examined. Here we assessed the diversity of AS events using web-based RNA-seq data of sorted CD15^-^CD11b^+^ microglia in white matter (WM) region from 10 patients with MS and 11 control subjects. The GSE111972 dataset was downloaded from GEO and ENA databases, aligned to the GRCh38 reference genome from ENSEMBL via STAR. rMATS was used to assess five types of AS events, alternative 3’SS (A3SS), alternative 5’SS (A5SS), skipped exon (SE), retained intron (RI) and mutually exclusive exons (MXE), followed by visualizing with rmats2sashimiplot and maser. Differential genes or transcripts were analyzed using the limma R package. Gene ontology (GO) analysis was performed with the clusterProfiler R package. 42,663 raw counts of AS events were identified and 132 significant AS events were retained based on the filtered criteria: 1) average coverage >10 and 2) delta percent spliced in (ΔPSI) >0.1. SE was the most common AS event (36.36%), followed by MXE events (32.58%), and RI (18.94%). Genes related to telomere maintenance and organization primarily underwent SE splicing, while genes associated with protein folding and mitochondrion organization were predominantly spliced in the MXE pattern. Conversely, genes experiencing RI were enriched in immune response and immunoglobulin production. In conclusion, we identified microglia-specific AS changes in the white matter of MS patients, which may shed light on novel pathological mechanisms underlying MS.

## INTRODUCTION

Alternative splicing (AS) is a pivotal transcriptional regulatory mechanism that arises from specific exons or introns that may or may not be included in a mature mRNA transcript. This process enables a single gene to produce multiple distinct mRNA molecules, thereby significantly increasing the diversity of the transcriptome and proteome [1]. AS is involved in a series of important biological processes, including proliferation, differentiation, development, and apoptosis regulation. The five common AS patterns are alternative 3’ splice site (A3SS), alternative 5’ splice site (A5SS), skipped exon (SE), retained intron (RI), and mutually exclusive exons (MXE) [2]. AS dynamically changes in different conditions, and aberrant alternative splicing can result in various neurological diseases, tumors, and is implicated in aging, infection, inflammation, immune disorders, and metabolic disorders [1].

Multiple sclerosis (MS) is a chronic neuroinflammatory disease characterized by demyelination and neuroaxonal damage, leading to the formation of lesions throughout the central nervous system (CNS) [3, 4]. Aberrant alternative splicing (AS) events have been documented in MS. The soluble form of the interleukin-7 receptor alpha chain gene (sIL7R), produced by alternative splicing of IL7R exon 6, is a driver of increased MS risk [5]. Aberrant splicing of exon 3B (DM20) of PLP could disrupt the PLP/DM20 ratio in thymic epithelial cells and lead to self-epitope rejection from central tolerance [6]; In an animal model of MS, SJL/J mice lacking thymic DM20 experience loss of central tolerance to this epitope and increased susceptibility to MS [7]. Several other SE and MXE events have also been well examined in MS [8–12].

As resident CNS macrophages, microglia play a crucial role in inflammatory lesions and associated neural dysfunctions [13]. Depletion of microglia has been shown in studies to provide significant neuroprotection in both the EAE and CPZ mouse models of MS, highlighting their detrimental role [14–19]. On the contrary, microglia also contribute to myelin repair through various mechanisms. They can phagocytose myelin, cell debris, and large cargoes [20], leading to cholesterol efflux and potentially promoting OPC proliferation and functions [21]. Microglia secretes trophic factors that support OPC proliferation and differentiation into myelinating oligodendrocytes [22]. Additionally, microglia engage in juxtacrine interactions with OPCs, potentially promoting specific signaling pathways [23]. The aforementioned opposing capabilities may be associated with the phenotypic heterogeneity of microglia.

AS can modulate the phenotype and function of cells [24, 25], however, the microglia-specific AS events in MS have not been extensively examined. Here we aimed to systematically assess AS events in sorted microglia (CD15^-^CD11b^+^) cells within the white matter of patients with MS and elucidated microglia-specific AS changes in the context of MS.

## MATERIALS AND METHODS

### Dataset and reference genome

GSE111972 was retrieved from the GEO database (https://www.ncbi.nlm.nih.gov/geo/query/acc.cgi?acc=GSE111972). The raw fastq files were downloaded from the ENA database using Linux via Axel (v2.17.11) (https://www.ebi.ac.uk/ena/browser/view/PRJNA438707). The human GRCh38 reference genome files were obtained from the ENSEMBL database. The DNA fasta file (https://ftp.ensembl.org/pub/release-107/fasta/homo_sapiens/dna/), cDNA fasta file (https://ftp.ensembl.org/pub/release-107/fasta/homo_sapiens/cdna/), and GTF file (https://ftp.ensembl.org/pub/release-107/gtf/homo_sapiens/) were downloaded for alignment and subsequent analysis.

### Alignment reading

Fastqc (v0.11.9) and multiqc (v1.13) were used for data quality control, trim_galore (v0.6.7) was used to clean data by setting parameters: trim_galore --phred33 -q 20 --length 36 --stringency 3 -a AGATCGGAAGAGC -o fastq-file, and then the clean data was aligned to the human reference GRCh38 genome by STAR (v2.7.10a) [26].

### Differentially expressed genes/transcripts identification

FeatureCounts and salmon were used to generate gene and transcript expression matrices, respectively. Differentially expressed genes/transcripts were determined using the Limma R package (v3.50.3), with a significance threshold set at a p-value of 0.05 and a log fold change (logFC) cutoff of 0.5. Unpaired Student’s t-test was employed to compare gene and transcript expression data between MS patients and controls.

### Alternative splicing analysis

After STAR process, we used the sorted bam files for AS analysis via rMATs v4.0.2 and then visualized them by rmats2sashiplot. We identified five types of differential AS events (A3SS, A5SS, SE, MXE, RI) between MS and control subjects, significant AS events were defined with strict filtering thresholds: 1) average coverage >10, 2) delta percent spliced in (∆PSI) >0.1 and 3) FDR < 0.05. The final statistics and visualization analysis were finished by maser, rtracklayer, echarts4r and ggplot2 R package.

### Gene ontology enrichment analysis

GO enrichment analysis of differentially expressed genes was conducted using the clusterProfiler R package. Visualization of the enrichment results was achieved through the Cnetplot function of the GOplot R package. Significant GO terms were determined based on a p-value threshold of < 0.05.

### Availability of data and materials

The dataset GSE111972 (https://www.ncbi.nlm.nih.gov/geo/query/acc.cgi?acc=GSE111972) was obtained from the Gene Expression Omnibus (GEO) database. All data generated in the current study can be found in the article and Supplementary Material.

## RESULTS

### Global summary of alternative splicing events

**Figure 1:**
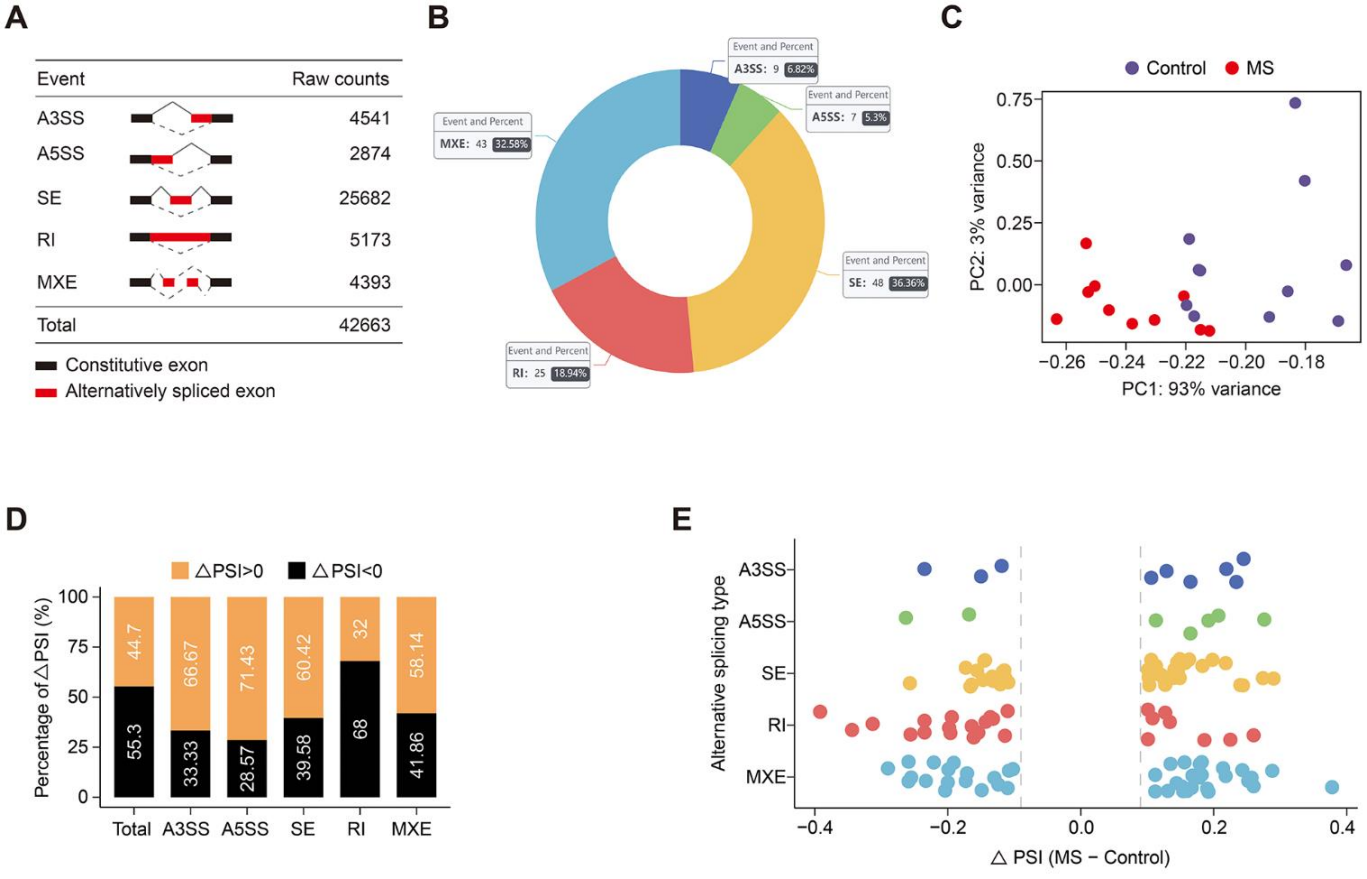
**Global distribution of alternative splicing events.** (**A**) Raw counts of each AS event. (**B**) The number and percentage of significant AS events. (**C**) PCA plot of each sample based on PSI values. (**D**, **E**) Bar (**D**) and dot (**E**) plots illustrate the ∆PSI distribution of each subtype and global AS events.

Altogether, 42,663 AS events were detected in the GSE111972 datasets (Figure 1A). Initially, we sought to identify differential AS events between MS and control subjects. We employed rMATS to quantify the percent spliced in (PSI) value for each AS event. Utilizing stringent criteria (average coverage > 10 and ∆PSI > 0.1), we identified, on average, 132 significant AS events (Figure 1B). Principal component analysis (PCA) of AS events effectively distinguished between MS and control groups (Figure 1C). PSI values were found to be elevated in A3SS, A5SS, SE, and MXE events, whereas RI and total AS events exhibited decreased PSI (Figure 1D, 1E). The top 2 events in each AS type are shown in Table 1, while all 132 significant AS events are listed in Supplementary Table 1.

**Table 1:** Top 2 events of each AS subtype.

**ID**	**Gene**	**p-value**	**FDR**	**∆PSI**	**Type**
**531**	CTSB	0	0	-0.023	A3SS
**1411**	IFRD1	0	0	-0.059	A3SS
**5973**	YWHAE	0	0	0.028	A5SS
**6458**	HNRNPA1	0	0	-0.037	A5SS
**424**	TKT	0	0	0.101	SE
**1957**	HSPH1	0	0	0.031	SE
**2766**	DDX3X	0	0	-0.069	RI
**8346**	GNAS	0	0	-0.039	RI
**715**	CD53	0	0	0.061	MXE
**1267**	CLK1	0	0	0.02	MXE

### Analysis of SE events

**Figure 2:**
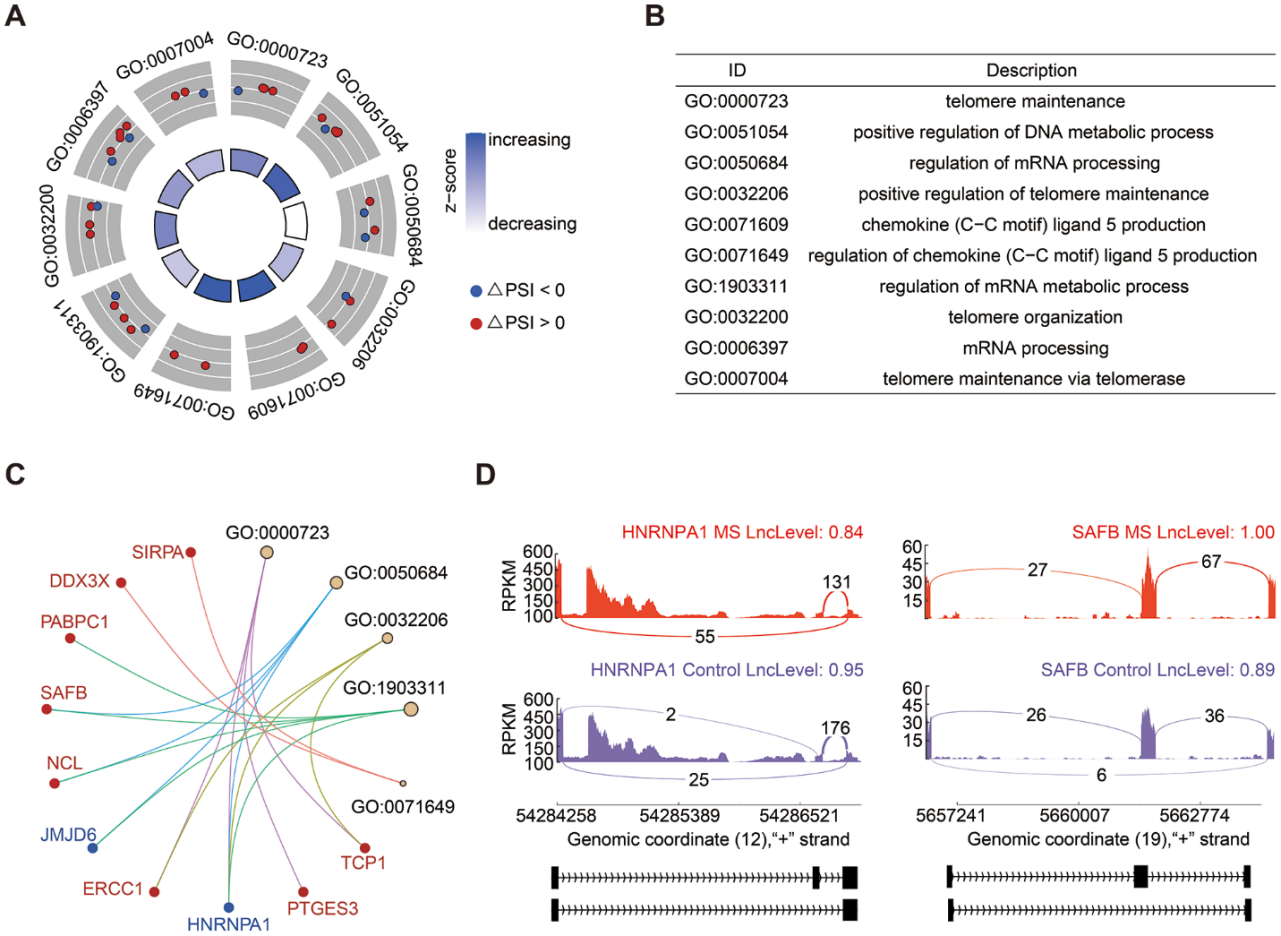
**Analysis of SE events.** (**A**, **B**) GO enrichment analysis of genes associated with SE events. (**C**) Cnetplot illustrating genes within enriched GO terms. HNRNPA1, highlighted in blue, exhibited decreased PSI levels, while SAFB, highlighted in red, demonstrated increased PSI levels. (**D**) Detailed sashimi plots depicting the splicing patterns of HNRNPA1 and SAFB. The red and purple colors denote the MS and control groups, respectively.

SE events emerged as the most prevalent alternative splicing events, constituting 36.36% of the total (Figure 1B). GO enrichment analysis of genes associated with significant SE events revealed enrichment in processes such as telomere maintenance and organization, regulation of mRNA processing, and metabolic processes (Figure 2A, 2B). Noteworthy genes implicated in these processes included Heterogeneous Nuclear Ribonucleoprotein A1 (HNRNPA1) and Scaffold Attachment Factor B (SAFB) (Figure 2C). The PSI value signifies whether an exon is included in the transcript quantification, essentially reflecting the exon length. In Figure 2D, it is evident that HNRNPA1 exhibited fewer exons, while SAFB displayed a tendency to have more exons in the MS group.

### Analysis of MXE events

**Figure 3:**
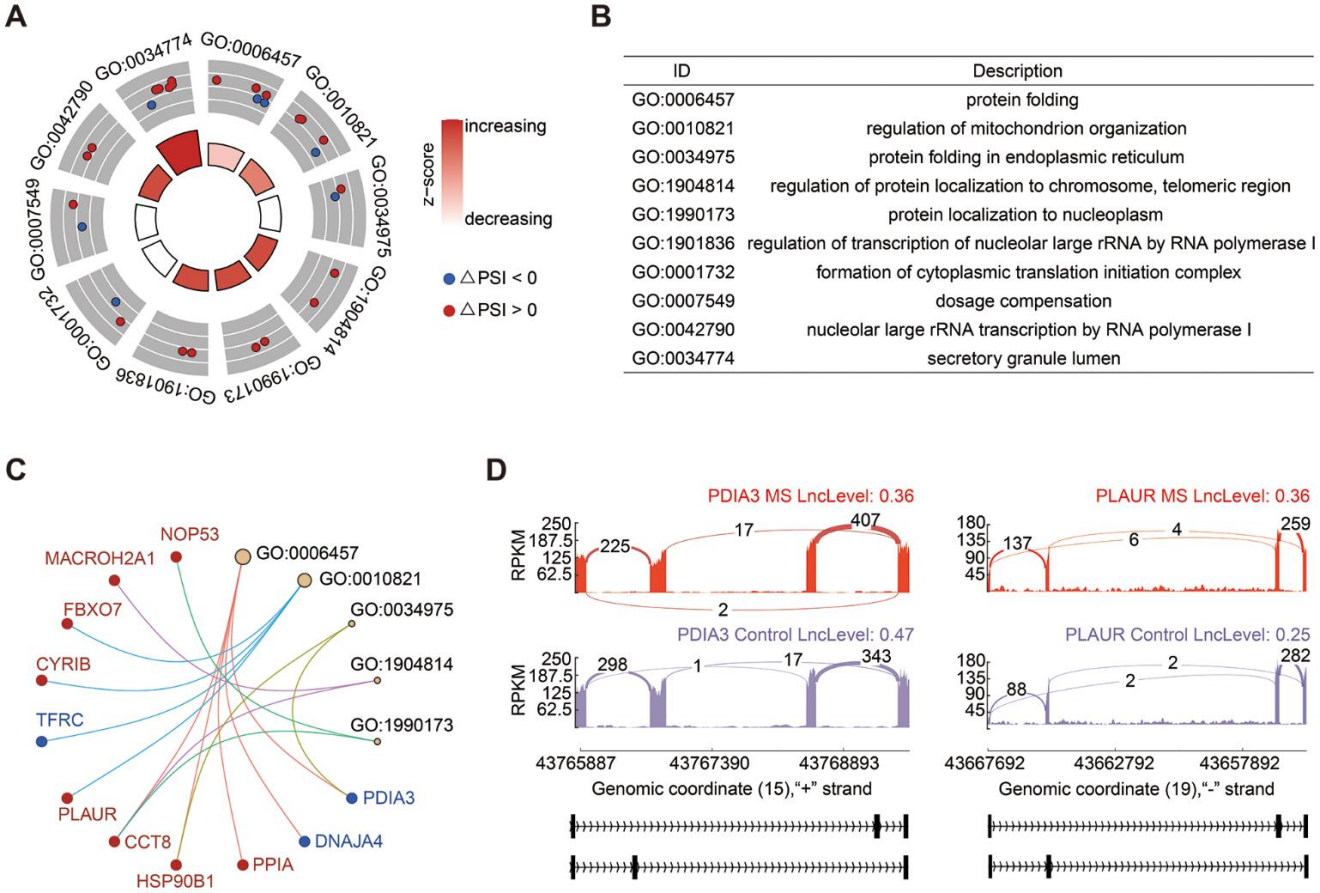
**Analysis of MXE events.** (**A**, **B**) GO enrichment analysis of genes involved in MXE events. (**C**) Cnetplot of genes in enriched GO terms. The PSI of PDIA3 was decreased and colored in blue, in contrast, PLAUR was colored in red. (**D**) Sashimi plots provided a detailed depiction of the alterations in transcripts of PDIA3 and PLAUR.

Mutually exclusive splicing represents a rare form of alternative splicing events, giving rise to alternative isoforms by selectively retaining only one exon from a cluster of neighboring internal exons. In our study, MXE exhibited the second most substantial changes in AS events, accounting for 32.58% (Figure 1B). GO analysis revealed significant enrichment in processes related to protein folding and mitochondrion organization (Figure 3A, 3B). Notable genes implicated in these processes included Protein Disulfide Isomerase Family A Member 3 (PDIA3), which exhibited a decreased PSI value, and Plasminogen Activator and Urokinase Receptor (PLAUR), which displayed an increased PSI (Figure 3C). Sashimi plots illustrated that PDIA3 tended to have shorter transcripts, whereas PLAUR exhibited longer transcripts in the MS group (Figure 3D).

### Analysis of RI events

**Figure 4:**
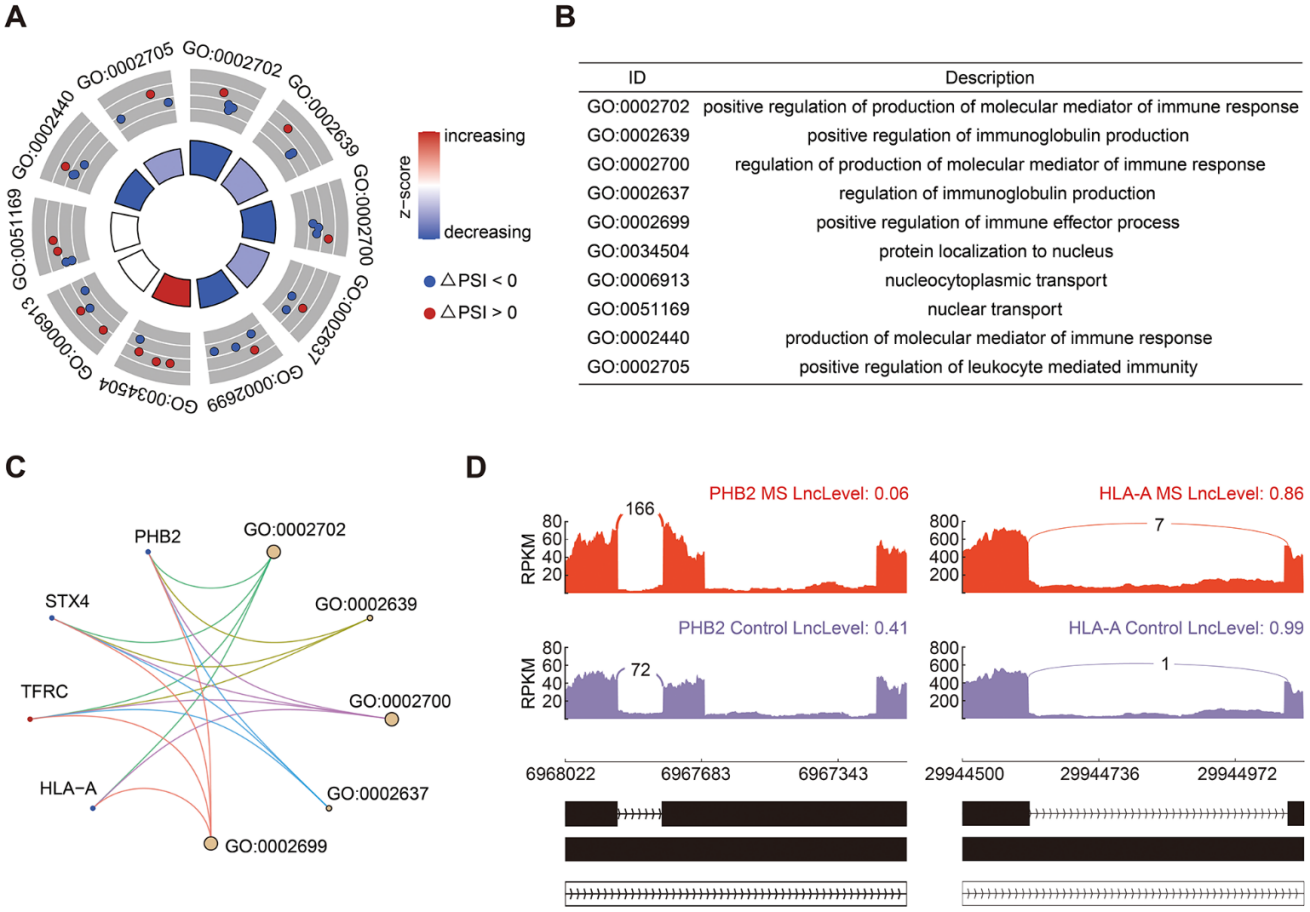
**Analysis of RI events.** (**A**, **B**) GO enrichment analysis of genes in RI events. (**C**) Cnetplot of genes involved in RI events. The decreased and increased PSI values were colored in blue and red, respectively. (**D**) Sashimi plots showed the detailed distribution of PHB2 and HLA-A transcripts.

A total of 25 significant RI events were identified, constituting 18.94% of all AS events. GO analysis of genes associated with RI events predominantly enriched in immune response and immunoglobulin production (Figure 4A, 4B), including genes of PHB2 (Prohibitin 2) and HLA-A (Major Histocompatibility Complex, Class I, A) (Figure 4C). The PSI value in there represents the inclusion level of retained intron. Consequently, both HLA-A and PHB2 exhibited shorter introns in the MS group (Figure 4D).

### Differentially expressed genes and transcripts

**Figure 5:**
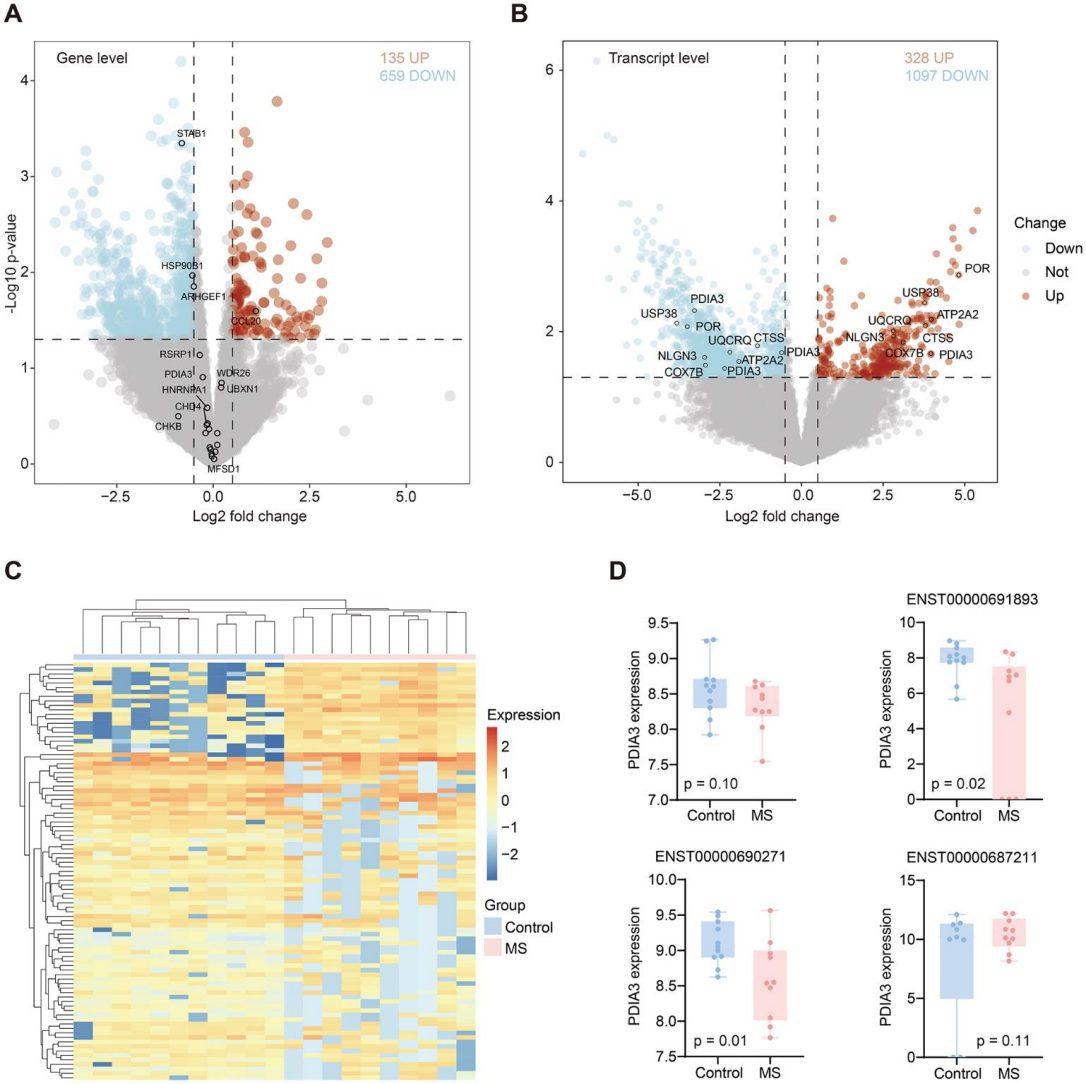
**Differentially expressed genes and transcripts.** (**A**) Differential genes volcano in MS compared with control group. Red and blue dots represent up and down-regulated genes, respectively. (**B**) Differential transcripts volcano in MS compared with control group. Red and blue dots represent up and down-regulated transcripts, respectively. (**C**) Heatmap displayed significantly different transcripts expression in MS and control group. (**D**) Boxplots showed the PDIA3 gene and its three transcripts’ differential expression between MS and control groups.

Transcript expression alterations did not consistently correlate with gene expression changes. Here, we investigated both differential gene and transcript expression levels. At the gene level, we identified 135 upregulated and 659 downregulated genes in the MS group compared with the control group (Figure 5A). Only four genes with significant AS events exhibited significant differences at the gene level. At the transcript level, we observed 328 upregulated and 1097 downregulated transcripts (Figure 5B). Heatmap illustrated the significant transcript expression differences between groups (Figure 5C). Among these transcripts, six genes with RI events were identified (Table 2). Several genes exhibited distinct transcript variations (ARHGEF1, CHD4, FCGRT, HSP90B1, PDIA3, STAB1, STXBP2). Notably, PDIA3 displayed contrasting expression changes across different transcripts; specifically, ENST00000691893 and ENST00000690271 presented lower expression levels, while ENST00000687211 exhibited a higher expression level in the MS group (Figure 5D).

**Table 2:** Dysregulated transcripts involved in significant AS genes.

**Transcript**	**LogFC**	**p-value**	**Gene**	**AS type**	**Change**
ENST00000594221	-2.443377412	0.037351933	STXBP2	A3SS	Down
ENST00000464225	-1.975566306	0.048896379	CHKB	A3SS	Down
ENST00000602355	-1.57576221	0.040415466	STXBP2	A3SS	Down
ENST00000698649	-3.415544757	0.011224147	IL6ST	A5SS	Down
ENST00000337665	-5.888629623	0.001529838	ARHGEF1	MXE	Down
ENST00000552051	-5.01562484	0.014368326	HSP90B1	MXE	Down
ENST00000691893	-3.27518664	0.004753761	PDIA3	MXE	Down
ENST00000550479	-3.08718845	0.020093518	HSP90B1	MXE	Down
ENST00000599988	-3.066989376	0.007804023	FCGRT	MXE	Down
ENST00000621632	-2.584937117	0.021911611	DDX24	MXE	Down
ENST00000687173	-2.353576829	0.036374286	PDIA3	MXE	Down
ENST00000482556	-0.669787702	0.022193598	PREX1	MXE	Down
ENST00000595881	-0.64643206	0.034239686	FCGRT	MXE	Down
ENST00000595897	-0.633561872	0.030335524	ARHGEF1	MXE	Down
ENST00000690271	-0.6058592	0.020887712	PDIA3	MXE	Down
ENST00000339082	1.625153521	0.049277156	PLAUR	MXE	Up
ENST00000486652	2.305175032	0.03466686	WDR26	MXE	Up
ENST00000687211	3.965479476	0.021572852	PDIA3	MXE	Up
ENST00000525717	-6.266450368	7.23E-07	UBXN1	RI	Down
ENST00000336783	-4.726381253	0.049410308	ATXN2L	RI	Down
ENST00000372862	-3.237874105	0.026721332	PABPC4	RI	Down
ENST00000642686	-3.107584683	0.008875577	CHD4	RI	Down
ENST00000481626	-2.418593172	0.031647809	STAB1	RI	Down
ENST00000479355	-2.404407387	0.015693043	STAB1	RI	Down
ENST00000647535	-2.071991228	0.047449398	CHD4	RI	Down
ENST00000461325	-0.93426492	0.005163376	STAB1	RI	Down
ENST00000462741	-0.846464911	0.009625139	STAB1	RI	Down
ENST00000321725	-0.807543744	0.000622462	STAB1	RI	Down
ENST00000471500	-2.958506153	0.016720016	MFSD1	SE	Down
ENST00000550482	-2.651282586	0.022632429	HNRNPA1	SE	Down
ENST00000532696	-1.850846736	0.023545454	DENND5A	SE	Down
ENST00000243189	-1.75741441	0.037455787	RSRP1	SE	Down
ENST00000456935	3.024758152	0.035062464	MAN2B1	SE	Up
ENST00000489160	3.818890414	0.002504536	CCL20	SE	Up

## DISCUSSION

Changes in AS events have been implicated in numerous diseases. Here, we investigated AS alterations in white matter microglia sorted by CD15 and CD11b from 10 MS patients and 11 control subjects. We identified five types of AS events, and genes implicated in various AS types exhibited unique biological functions. Despite several reported aberrant AS events in MS, we, for the first time, explored cell type-specific AS changes in MS.

Consistent with prior investigations, SE events were the most prevalent AS events, constituting 36.36% of occurrences [27–29], followed by MXE events at 32.58%, and RI events at 18.94%, while A3SS and A5SS events comprised 12.12%. GO enrichment revealed that genes involved in significant SE events were primarily associated with biological processes such as telomere maintenance and organization, regulation of mRNA processing, and metabolic processes. Notably, this included genes like HNRNPA1 and SAFB. HNRNPA1 encodes a member of the ubiquitously expressed heterogeneous nuclear ribonucleoprotein (hnRNP) family, whose RNA-binding proteins play a pivotal role in alternative splicing regulation. Mutations in HNRNPA1 have been identified in individuals with MS, potentially exacerbating neurodegeneration in this context [30]. Our findings indicate a shorter transcript of HNRNPA1 in MS. On the other hand, SAFB encodes the essential protein SAFB1, which governs gene regulation in the brain. Aberrant expression of SAFB has been documented in the nerve cells of brain regions affected by spinocerebellar ataxias (SCAs) and Huntington’s disease (HD), where it is associated with specific polyglutamine expansion pathology. Our study unveils a longer transcript of SAFB in MS.

MXE is a rare subtype of AS events. In the splicing of MXE, two (or more) splicing events are no longer independent but are executed or disabled in a coordinated manner [31]. It ranked as the second most prevalent AS event in our study. Genes associated with MXE events were primarily enriched in processes related to protein folding and mitochondrion organization, featuring prominent candidates such as PDIA3 and PLAUR. Prior research has suggested that the reduction of PDIA3 expression/activity in glioblastoma cells can significantly restrain microglia pro-tumor polarization towards the M2 phenotype and the production of pro-inflammatory factors [32]. Our investigation revealed that PDIA3 exhibited shorter exons in MS; however, its overall gene expression did not change significantly. PLAUR has also been implicated in Glioma and is suggested to play roles in immunosuppression [33]. Interestingly, it demonstrated a tendency to have longer exons in MS. These two genes might be linked to alterations in microglial function in the context of MS and warrant further in-depth investigations.

RI stands out as the third most prevalent subtype of alternative splicing (AS) events. While a substantial portion of transcripts arising from RI events undergo degradation via nonsense-mediated decay (NMD), a subset retains translational potential [34]. Our analysis identified 25 RI events, with the associated genes primarily implicated in biological processes related to immune response and immunoglobulin production. Noteworthy genes within this category include PHB2 and HLA-A. Immune-related genes, particularly those encoding human leukocyte antigen (HLA), exhibit a diversity of spliced isoforms. Previous research has reported that HLA-A*03 confers an increased risk for multiple sclerosis (MS), whereas HLA-A*02 exerts a protective effect [35]. Therefore, alternative splicing within the HLA-A gene may also be linked to the risk of MS. In the context of MS, both PHB2 and HLA-A displayed shorter transcripts. While an elevation in PHB2 protein levels in peripheral blood mononuclear cells (PBMC) has been documented [36], it is disappointing that we did not observe a significant alteration at the gene level for PHB2 in MS compared with control subjects.

AS can lead to changes in the expression levels of both genes and transcripts, although these changes are not always consistent due to the tight regulation of AS itself. In our study, we identified 135 upregulated and 659 downregulated genes in MS. However, only four genes (STAB1, HSP90B1, ARHGEF1, CCL20) exhibited significant changes at the gene level within significant AS events. Notably, PDIA3 demonstrated control over different transcripts with opposite changes at the transcript level, which is of particular interest. This suggests that AS may influence transcript expression prior to any observable changes at the gene expression level.

Together, our studies uncovered microglia-specific AS changes in MS. SE genes were found to be enriched in telomere function, MXE genes were implicated in protein and mRNA processing, and RI genes were associated with immune response. Collectively, these data may shed light on novel pathological mechanisms underlying MS.

## Supplementary Material

Supplementary Table 1
